# A cross-sectional study to assess knowledge about HIV/AIDS transmission and prevention measures in company workers in Ecuador

**DOI:** 10.1186/1471-2458-13-139

**Published:** 2013-02-15

**Authors:** María C Cabezas, Marco Fornasini, Nadia Dardenne, Teresa Borja, Adelin Albert

**Affiliations:** 1 Psychology School, Universidad San Francisco de Quito (USFQ), Av. Diego de Robles y Vía Interoceánica, Cumbaya, Quito, Ecuador; 2 Translational Research Center, Universidad de las Américas (UDLA), Av. de los Granados y Colimes, Quito, Ecuador; 3 Public Health Department, University of Liège, Sart Tilman, B23, 4000, Liège, Belgium; 4 Department of Medical Informatics and Biostatistics, University of Liège, Sart Tilman, B23, 4000, Liège, Belgium

**Keywords:** HIV/AIDS, Ecuador, Prevention, Transmission, Educational

## Abstract

**Background:**

HIV/AIDS was first reported in Ecuador in 1984 and its prevalence has been increasing ever since. In 2009, the National AIDS Program reported 21,810 HIV/AIDS cases and confirmed that the worker population was amongst the most affected groups. The objective of this study was to assess knowledge about HIV transmission and prevention measures in company workers in Ecuador.

**Methods:**

A cross-sectional survey based on a random sample of 115 companies (1,732 workers), stratified by three large provinces and working sectors (commerce, manufacturing and real estate) was conducted. A validated instrument developed by Family Health International was used to evaluate HIV prevention knowledge and common local misconceptions about HIV transmission. Descriptive statistics, chi square test and logistic regression analysis were performed using SAS.

**Results:**

Incorrect knowledge about HIV/AIDS transmission were found in 49.1% (95% CI: 46.6–51.6) of subjects. Incorrect knowledge was higher among males (OR = 1.73 [1.39–2.15]), older subjects (OR = 1.35 [1.02–1.77]), subjects with lower education (OR = 3.72 [2.44–5.65]), manual labor workers (OR = 2.93 [1.82–4.73]) and subjects without previous exposure to HIV intervention programs (OR = 2.26 [1.79–2.86]). Incorrect knowledge about preventive measures was found among 32.9% (95%CI: 30.6–35.2) of respondents. This proportion was higher among subjects with lower education (OR = 2.28 [1.52–3.43]), married subjects (OR = 1.34 [1.07–1.68]), manual labor workers (OR = 1.80 [1.34–2.42]), and subjects not previously exposed to HIV intervention programs (OR = 1.44 [1.14–1.83]).

**Conclusions:**

HIV intervention programs targeting company workers are urgently needed to improve knowledge and reduce HIV transmission in Ecuador.

## Background

HIV/AIDS is still a major epidemic worldwide, despite considerable advances in the diagnosis, treatment and prevention of the disease over the last 20 years. The first cases of HIV/AIDS were reported in the early eighties in Los Angeles by the Center for Disease Control and Prevention (CDC) [1]. According to the UNAIDS 2009 report, some 60 million people have been infected worldwide since the start of the epidemic, nearly 30 million people have died of HIV-related causes and there are 14 million children orphaned due to HIV deaths in Southern Africa alone [2]. In 2009, the same report cited an estimated number of 2.6 million people newly infected with HIV [3]. At the end of 2010, more than 34 million people were reported to live with HIV. The disease was considered to be pandemic by the CDC as of August 2006. Today, sexual intercourse and needle exchange are the two main causes of HIV transmission. Understanding the health problem while also creating prevention programs to promote safe sex and safe needle exchange-programs are challenging issues in every country impacted by the epidemic.

In South and Central America, the epidemic has evolved in recent years. The total number of people living with HIV continued to grow to an estimated 1.4 million in 2009 and about 92,000 new HIV infections were reported during the same year [4]. Ecuador is a middle-income country with health issues related to poverty, low education levels, gender inequality, religious restrictions and race discrimination. The first case of HIV/AIDS in Ecuador was reported in 1984 by the Ministry of Public Health of Ecuador (MPHE). In August 2007, the MPHE registered 12,246 people infected with HIV. Most cases were found among the worker population, among subjects 20–44 years old, and in the heterosexual population (80.1%) [5]. During 2008, the MPHE reported 15,000 HIV/AIDS positive cases. The age group most affected was the 20–35 year-old, which is a very productive age of life. In addition, 99% of the infections were due to sexual contact [6].

**Figure 1:**
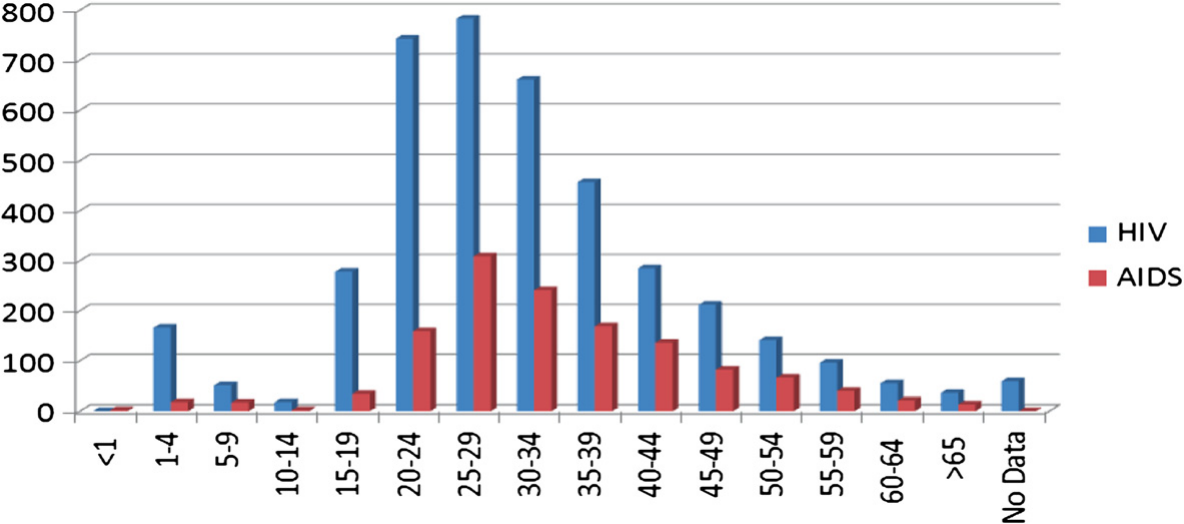
**Distribution of HIV/AIDS cases per age group in Ecuador in 2009. **(Source: Ministerio de Salud Pública del Ecuador. Programa Nacional de SIDA).

In 2007, an under-registry of HIV positive cases in Ecuador was identified by UNAIDS; this organism estimated that more than 40,000 persons infected with HIV in Ecuador were not aware of their infection and hence at risk of transmitting HIV to others [7]. It is therefore a priority to identify HIV-infected persons and to link them to medical, prevention, and other services as soon as possible after they become infected [8]. In 2009, the number of HIV/AIDS cases registered in Ecuador rose to 21,810 and the same age group was most impacted as in previous reports [9]. In 2010, according to the National AIDS Program Director in Ecuador, the number of HIV cases had been increasing steadily for the past 25 years and workers were among the groups most affected by HIV infection (see Figure 1).

With regards to the routes of HIV transmission in Ecuador, 99% of the positive cases were due to sexual activities [6]. HIV testing is essential to avoid the spread of infection, although infected people can still transmit the virus during the window period. In the last year, a significant increase of HIV/AIDS cases was reported most likely due in part to the implementation of HIV testing in pregnant women, blood donors, positive tuberculosis cases and sexually transmitted disease consultations [9]. This testing increased the prevalence of HIV cases in the population of pregnant women but facilitated treatment access in newborns and pregnant women according to the last UNGASS Report [9]. It should be noted that information about the number of HIV tests done every year among the entire Ecuadorian population is not yet available. In fact, Ecuador suffers from a lack of research findings concerning HIV/AIDS and the limited information available differs greatly from official statistics released by the Ministry of Public Health of Ecuador. Overall, the actual magnitude of the epidemic and its risk of spreading in the country are hardly known, despite an increasing number of initiatives among young researchers [9].

According to the World Health Organization (WHO), the noteworthy drop in HIV incidence worldwide has been associated (in both genders) with several behavior indicators, including increased condom use, delayed sexual initiation, and reduction in multiple partnerships. Hence, it is believed that the most important factors to control the spread of the infection are the behavioral changes towards sexuality which can best be triggered by improving basic knowledge about HIV/AIDS transmission pathways and prevention measures. Estimating knowledge about HIV in the general population is a difficult task. It can be done on specific subgroups of individuals at risk but these groups may not be easily accessible. It can also be done locally or in restricted areas, but results can hardly be extrapolated to larger populations. By contrast, companies which employ large portions of the sexually active population are structured and recognized entities. Their workers have themselves a life outside the occupational setting: family, friends, and social activities. Thus, assessing HIV/AIDS knowledge among workers of companies may have a far wider impact than in other settings.

The present research study was designed to estimate by means of a validated questionnaire the prevalence of HIV/AIDS transmission and prevention measures knowledge among the population of workers from three working sectors (commerce, manufacturing, and real estate) in three provinces (Pichincha, Guayas and Azuay) of Ecuador. These provinces were selected because they are large, representative of the country and host most of the Ecuadorian companies. As for the three working sectors selected, they encompass the largest number of companies and also the greatest number of workers.

## Methods

### Study design

This cross-sectional study was based on a random sample of 115 companies stratified by province (Pichincha, Guayas and Azuay) and working sector (commerce, manufacturing and real estate). In each stratum, the companies were selected randomly and proportionally to the actual number of companies recorded in the database of the “Superintendencia de Companías” of Ecuador (see Table 1). The number of companies recorded in the database for the selected provinces and working sectors was 34,917 and the corresponding number of workers was 311,050. In the database, workers are also classified into job categories (executive, administrative, manual labor and other).

**Table 1:** Number of companies by province and working sector in the population (sample)

**Province**	**Working sector**				
	**Commerce**	**Manufacturing**	**Real Estate**	**Total**	**%**
Pichincha	5528 (18)	1806 (6)	5608 (19)	12942 (43)	37.1
Guayas	7833 (26)	1912 (6)	10727 (35)	20472 (67)	58.6
Azuay	721 (2)	289 (1)	493 (2)	1503 (5)	4.3
Total	14082 (46)	4007 (13)	16828 (56)	34917 (115)	100
%	40.0	11.3	48.7	100	

Workers were selected proportionally to the number of workers by working sector and job category (see Table 2). This was achieved by selecting groups of approximately 15, 45 and 10 workers from each company of the commerce, manufacturing and real estate working sector, respectively, and keeping a fair representation of job categories. The actual selection of workers proceeded as follows. For each company, the number of workers was known but no lists of names were used to guarantee anonymous selection. The company was contacted by mail or phone to participate in the study, and a formal invitation letter with information about the study was sent to the General Manager and Human Resources Manager. In case of refusal, another company was drawn from the database. When the company agreed to participate, workers were invited to attend a free information meeting on HIV/AIDS, where the study and its objectives were explained. After signing the informed consent form to confirm participation in the study, participants were asked to fill in the “Family Health International” questionnaire. All questionnaires were collected by the organizers, kept anonymous and sorted by job category. In order to maintain the sample size initially planned for the company and the proportions of job categories, questionnaires in excess were discarded randomly. This procedure was applied in all 115 companies, yielding a total number of 1732 workers (see Table 2). The distribution of workers according to province was: Pichincha (n = 736, 42.5%), Guayas (n = 900, 52.0%) and Azuay (n = 96, 5.5%).

**Table 2:** Number of workers by working sector and job category in the population (sample)

**Job category**	**Working sector**				
	**Commerce**	**Manufacturing**	**Real Estate**	**Total**	**%**
Executive	6732 (38)	2869 (16)	6356 (35)	15957 (89)	5.1
Administrative	38054 (212)	20806 (116)	17966 (100)	76826 (428)	24.7
Manual Labor	37107 (206)	70770 (394)	48139 (268)	156016 (868)	50.2
Other	30197 (168)	11572 (64)	20485 (115)	62254 (347)	20.0
Total	112090 (624)	106017 (590)	92946 (518)	311050 (1732)	100
%	36.0	34.1	29.9	100	

### Subject characteristics

For each participant, the following sociodemographic variables were collected: age (years), gender, education level (primary, secondary, higher), marital status (married, single), job category (executive, administrative, manual labor, other) and previous exposure to an HIV/AIDS educational intervention program (yes, no). Age was dichotomized as <40 or ≥40 years. Data were also collected on the subject’s sexual activity, specifically sexual relations over the past 12 months (yes, no) with a regular partner, a commercial partner, and an occasional partner. In each case, the estimated number of partners was noted as well as condom use or lack thereof.

### Questionnaire and study conduct

The instrument used in this study was the “Behavioral Surveillance Surveys – Adult questionnaire” developed by Family Health International in 2000. This questionnaire includes 18 questions about knowledge, opinions and attitudes towards HIV/AIDS [10]. Before implementing the survey, all questions were validated regarding language and comprehension of questions by a pilot study in Ecuador. Questionnaires were self-administered with facilitator help (mean time of 30–45 minutes). A surveyor manual was elaborated with the help of a sociologist to avoid bias in the survey process. The study was conducted by 10 surveyors (4 in Pichincha, 4 in Guayas and 2 in Azuay), 2 call centers and one study coordinator. The surveyors went through a two-day training session during which they were provided with information regarding the questionnaire and the project, and their questions were answered. Call centers staff received a one-day full time training. Validated informed consent forms explaining the study procedures, including protocols for subject confidentiality, were signed by each participant. Electronic data records were kept anonymous and secured.

### Measurement methods

Knowledge regarding HIV transmission was evaluated, along with the most common local misconceptions about HIV transmission, using questions Q904, Q907 and Q909:

• Q904: Can a person get HIV from mosquito bites?

• Q907: Can a person get HIV by sharing a meal with someone who is infected?

• Q909: Do you think that a healthy-looking person can be infected with HIV, the virus that causes AIDS?

Correct answers to all 3 questions defined “good knowledge” about disease transmission, otherwise the subject was declared to “lack knowledge about HIV/AIDS transmission”. Subjects with all three missing answers were not included in the statistical analysis.

Prevention measure knowledge was evaluated by means of questions about consistent condom use and mutual monogamy. These included questions Q903 and Q905:

• Q903: Can people protect themselves from the virus that causes HIV/AIDS by using a condom correctly every time they have sex?

• Q905: Can people protect themselves from HIV by having one uninfected faithful sex partner?

Correct answers to both questions defined “good knowledge” about the prevention measures, otherwise the subject was declared to “lack knowledge about HIV/AIDS prevention measures”. Subjects with both missing answers were not included in the statistical analysis.

### Ethics

The study was approved and controlled by The Institutional Review Board (IRB) of Universidad San Francisco de Quito in Ecuador.

### Statistical analysis

For practical reasons, a maximum of 115 companies was involved in the study. A power calculation showed that with a sample size of at least 1,500 workers, the prevalence could be estimated with a statistical precision of at least 3%. Quantitative variables were summarized by median and interquartile range (IQR), or by mean and standard deviation (SD), whereas for qualitative variables the number and percentage of subjects in each category were given. Mean values were compared by one-way analysis of variance and proportions by the chi-square test. The prevalence of “lack of knowledge” was estimated and associated with a 95% confidence interval (95%CI). The agreement between the knowledge assessments on transmission and on prevention measures was estimated by Cohen’s kappa coefficient; the closer the value to 1, the better the concordance.

The association between each risk factor and knowledge prevalence was assessed by the odds ratio (OR) and associated 95% CI adjusted for the stratifying variables (province and working sector). All risks factors and stratifying variables were then combined into a multivariate logistic regression analysis to account for potential confounders. The multivariate strength of association between lack of knowledge and the risk factors was measured by the area under the ROC curve (AUC) expressed in percent. Results were considered statistically significant at the 5% critical level (P < 0.05). All calculations were done using the SAS statistical package (version 9.2 for Windows).

## Results

### Description of the study participants

A total of 1,732 subjects were included in the study. Their characteristics are described in Table 3. The median age of the participants was 31 years (IQR: 15–69) and 1,374 (82.9%) of the participants were under 40 years of age. There were 1,128 (65.1%) men and 604 (34.9%) women. A majority of the participants (n = 929, 58.7%) were married. The education level was primary for 10.6%, secondary for 32.7% and higher for 56.7% of the participants. The job category of the participants was distributed as follows: 89 (5.1%) executives, 428 (24.7%) administrative personnel, 864 (50.1%) manual labor workers and 347 (20.1%) with other jobs. Previous exposure to an HIV/AIDS intervention program was high: 1,103 (67.9%) participants had been exposed and 522 (32.1%) had not. Among the former, the prevention program was given during the last year for 24.6% of the participants, 1–2 years ago for 29.4% and more than 2 years ago for 46.1%.

**Table 3:** Baseline population characteristics (n = 1732 Ecuadorian workers)

**Variable**	**Category**	**Number**	**%**
**Gender**	Male	1128	65.1
	Female	604	34.9
Age (years)*	<40	1374	82.9
	>40	284	17.1
**Education Level**	Primary	177	10.6
	Secondary	546	32.7
	Higher	947	56.7
**Marital Status**	Married	929	58.6
	Single	655	41.4
**Job Category**	Executive	89	5.1
	Administrative	428	24.7
	Manual Labor	868	50.1
	Other	347	20.1
**HIV Program Exposure**	Yes	1103	67.9
	No	522	32.1
**Sexual Relations**	Regular partner	1421	91.0
	Commercial partner	311	19.9
	Occasional partner	489	31.3
**Condom Use**	Regular partner (N = 1314)	282	19.9
	Commercial partner (N = 238)	177	56.9
	Occasional partner (N = 391)	168	34.4

*Median age 31 (IQR: 15–69) years.

Sexual relations over the past 12 months with a regular partner were recorded in 1,421 (91%) subjects, condom use was reported by 19.9% of subjects and most participants (65.5%) had only one regular partner. For sexual relations with commercial partners, 311 (19.9%) of the participants responded positively, condom use was reported in 56.9% of those cases, and the percentage of cases with more than one sexual partner was 73.5%. Sexual relations with an occasional partner were reported by 489 (31.3%) participants, with condom use noted in 34.4% of those cases and the number of occasional partners equal to one in 54.7% of the cases reported.

When comparing workers characteristics according to province and working sector marked differences were found (data not shown) but these differences were taken into account by adjusting for the two stratification factors in the subsequent analyses.

### Knowledge about HIV transmission

The overall prevalence of incorrect beliefs about HIV/AIDS transmission was based on 1,543 (89.1%) subjects from the initial sample. Among these subjects, 757 missed at least one correct answer, yielding a point prevalence of a lack of knowledge about HIV/AIDS transmission of 49.1% (95% CI: 46.6–51.6). The prevalence markedly varied according to province (Pichincha 51.1%, Guayas 45.4% and Azuay 66.7%, P = 0.0004). It was also different between working sector (commerce 49.2%, manufacturing 56.6%, and real estate 40.4%, P < 0.0001). When considering each risk factor separately and adjusting for province and working sector (see Table 4), the prevalence was found to be higher in older than in younger individuals, the adjusted OR being 1.35 (95%CI: 1.02–1.79). Furthermore, male gender was also significantly associated with a higher prevalence of lack of knowledge (adjusted OR = 1.73; 95%CI: 1.39–2.15) and so were education level (OR = 3.72; 95%CI: 2.44–5.65 for primary vs. higher), manual labor (OR = 2.93; 95%CI: 1.82–4.72) and non-exposure to a previous educational intervention program (OR = 2.26; 95%CI: 1.79–2.87). By contrast, no difference was seen for marital status (OR = 1.20; 95%CI: 0.97–1.48). When combined into a multivariate regression analysis, all previous factors but age (P = 0.079) remained significantly associated with misconceptions of HIV/AIDS transmission pathways after adjusting for the stratification variables. The AUC of the ROC curve quantifying the multivariate association between prevalence and risk factors amounted 70.2%.

**Table 4:** Prevalence and risk factors of lack of knowledge about HIV/AIDS transmission

**Variable**	**Category**	**N**	**Lack of knowledge**	**OR (95% CI)**^**(a)**^	**P–value**^**(a)**^	**P–value**^**(b)**^
			**Number (%)**			
Prevalence		1543	757 (49.1)			
Age (years)	< 40	1246	596 (47.8)	1.0	0.035	0.11
	≥ 40	243	136 (56.0)	1.35 (1.02–1.77)		
Gender	Female	556	228 (41.8)	1.0	< 0.0001	0.0005
	Male	997	529 (53.1)	1.73 (1.39–2.15)		
Educational level	Primary	131	94 (71.7)	3.72 (2.44–5.65)	< 0.0001	0.0009
	Secondary	480	310 (64.6)	2.87 (2.26–3.64)		
	Higher	883	330 (37.4)	1.0		
Marital Status	Married	821	413 (50.3)	1.20 (0.97–1.48)	0.10	0.24
	Single	604	278 (46.0)	1.0		
Job Category	Executive	84	30 (35.7)	1.0	< 0.0001	0.026
	Administrative	400	151 (37.8)	1.16 (0.70–1.90)		
	Manual labor	737	444 (60.2)	2.93 (1.82–4.73)		
	Other	322	132 (41.0)	1.47 (0.88–2.45)		
HIV Program Exposure	Yes	1038	438 (42.2)	1.0	< 0.0001	< 0.0001
	No	452	292 (64.6)	2.26 (1.79–2.86)		

^(a)^Univariate logistic regression but adjusted for province and working sector.

^(b)^Multivariate logistic regression adjusted for province and working sector (N = 1320) – AUC = 70.2%.

### Knowledge about HIV prevention measures

The overall prevalence of misconceptions regarding HIV/AIDS prevention measures was based on 1,546 (89.3%) subjects of the initial sample. Among these subjects, 509 missed at least one correct answer, yielding a point prevalence of 32.9% (95%CI: 30.6–35.2). The prevalence was comparable in the various provinces (Pichincha 30.4%, Guayas 34.2% and Azuay 40.5%, P = 0.098) and in the different working sector (commerce 34.6%, manufacturing 34.7%, and real estate 29.0%, P = 0.096). When considering risk factors separately and adjusting for province and working sector (see Table 5), age and gender were not significantly related to lack of knowledge. The prevalence in older subjects was 28.8% vs. 33.2% in younger individuals (P = 0.18), yielding an adjusted OR of 1.23 (95%CI: 0.91–1.68). As for gender, there was only a slight tendency (P = 0.071) towards a higher prevalence of a lack of knowledge in men (34.4%) than in women (30.2%): adjusted OR = 1.23 (95%CI: 0.98–1.55). By contrast, the education level was a significant risk factor for the lack of knowledge about HIV/AIDS prevention measures (P < 0.0001), with an adjusted OR for primary vs. higher equal to 5.33 (95%CI: 3.25–8.77). Similarly, the prevalence was significantly higher in manual labor (39.7%) than in others (25.7%) (adjusted OR = 1.80, 95%CI: 1.34–2.42). Married status (adjusted OR = 0.75, 95%CI: 0.60–0.93) and exposure to a previous HIV intervention program (adjusted OR = 0.69, 95%CI: 0.55–0.88) were clearly protective factors. When combining all factors into a multivariate logistic regression analysis, age (P = 0.040), education level (P = 0.0010), marital status (P = 0.0017) and exposure to a previous intervention program (P = 0.034) were all significantly related to lack of knowledge on HIV/AIDS prevention measures after adjusting for stratification and profession was no longer significant (P = 0.14). The AUC under the ROC curve was equal to 63.4%, slightly lower than for the knowledge prevalence about HIV transmission.

**Table 5:** Prevalence and risk factors of lack of knowledge about HIV/AIDS prevention measures

**Variable**	**Category**	**N**	**Lack of knowledge**	**OR (95% CI)**^**(a)**^	**P–value**^**(a)**^	**P–value**^**(b)**^
			**Number (%)**			
Prevalence		1546	509 (32.9)			
Age (years)	< 40	1250	415 (33.2)	1.24 (0.91–1.68)	0.17	0.038
	≥ 40	240	69 (28.8)	1.0		
Gender	Female	546	165 (30.2)	1.0	0.071	0.33
	Male	1000	344 (34.4)	1.23 (0.98–1.55)		
Educational level	Primary	123	53 (43.1)	2.28 (1.52–3.43)	< 0.0001	0.0042
	Secondary	479	200 (41.8)	2.06 (1.61–2.62)		
	Higher	893	236 (26.4)	1.0		
Marital Status	Married	824	248 (30.1)	1.0	0.011	0.017
	Single	607	218 (35.9)	1.34 (1.07–1.68)		
Job Category	Executive	82	28 (34.2)	1.40 (0.83–2.38)	< 0.0001	0.16
	Administrative	405	106 (26.2)	0.99 (0.70–1.38)		
	Manual Labor	736	292 (39.7)	1.80 (1.34–2.42)		
	Other	323	83 (25.7)	1.0		
HIV Program Exposure	Yes	1049	316 (30.1)	1.0	0.0025	0.039
	No	449	174 (38.8)	1.44 (1.14–1.83)		

^(a)^Univariate logistic regression but adjusted for province and working sector.

^(b)^Multivariate logistic regression adjusted for province and working sector (N = 1327) – AUC = 63.4%.

### Combined knowledge about HIV

When cross-classifying HIV/AIDS transmission knowledge with HIV/AIDS prevention measure knowledge, only 560 subjects over 1,494 (35.7%) responded correctly to all questions and 277 (18.5%) subjects failed on both knowledge issues. In other words, 64.3% lacked knowledge about at least one of the two aspects, quite a high prevalence. Cohen’s kappa coefficient measuring the agreement between transmission knowledge and prevention measure knowledge was found to be 0.11 (95%CI: 0.062-0.16), indicating poor concordance.

## Discussion

The present study is part of a larger three-phase project to improve the awareness about HIV/AIDS transmission pathways and prevention measures in occupational settings in Ecuador. Several national reports have persistently indicated that the company environment has been a major factor in the spread of the disease in the country. The present work reports on the first phase of the project, namely to estimate HIV/AIDS transmission and prevention measures knowledge among company workers by means of a cross-sectional study in three provinces and three working sectors. Based on the outcome of that study, a second phase is planned to develop an educational prevention program specifically designed for companies. The third phase is intended to assess the impact of the educational intervention phase on workers’ knowledge about HIV/AIDS.

To estimate the prevalence of transmission knowledge, three questions from the “Behavioral Surveillance Surveys – Adult questionnaire” were selected which provide pertinent information on the misconception of how the disease actually spreads in the population as demonstrated by another study [10]. In addition, prevention measures knowledge was evaluated by means of consistent condom use and mutual monogamy, the most important primary ways of avoiding HIV infection among sexually active men and women. The indicator of prevention measures knowledge is particularly useful in countries where knowledge is not high (as in Ecuador) to evaluate an educational intervention impact [10]. According to the 2011 UNAIDS Report, the effectiveness of an HIV intervention program is strongly related to improved knowledge and practice of preventive measures relating to HIV and sexually transmitted infections in the general adult population as well as in youth and vulnerable population sub-groups [11]. The present study used the original version of the Family Health International questionnaire. All subsequent versions of this questionnaire developed after 2000 maintained the same basic concepts and included modifications based mainly on local additions rather than altering significantly the established questions.

Clearly the proportion of workers with a lack of knowledge about HIV/AIDS in this study was high: 46.6% for transmission of the disease and 32% for prevention measures, respectively. Only 35.7% of the participants answered all five questions correctly. Risk factors were almost the same, namely male gender, older age, low education level and manual labor. By contrast, married status and previous exposure to an intervention program were protective and associated with better knowledge. The results of the “HIV/AIDS Linea de base” study sponsored by Global Fund in Ecuador in 2007 which focused on incorrect beliefs about forms of HIV transmission among men who have sex with men (MSM) and sex workers, were similar to those of the present study (good transmission knowledge: MSM 58.8% and sexual workers: 46.5%) [5]. The main objective of that study was to establish the prevalence of HIV/AIDS among MSM and sex workers. The results regarding HIV prevalence were interpreted cautiously because of potential biases in the selection methods [9]. The results regarding knowledge, however, were accepted and published by the Public Health Ministry of Ecuador (MPHE). Another study, the “Equidad Study” in 2006 (n = 261), revealed similar trends but was also conducted among the MSM and sex worker population and included only 2 questions about transmission knowledge (condom use: 78.2% and fidelity: 64.8%). Other studies conducted by the Ministry of Public Health of Ecuador to estimate the prevalence of HIV, namely in college students (ESPOCH, 2007), in sex workers (Red Trab Sex, 2007) and in MSM (CEPAR 2009) are worth mentioning but provide limited information on the topics discussed here [9]. To the best of our knowledge, there are no studies that have focused on the working population.

This is the first study carried out in Ecuador focusing on HIV/AIDS knowledge in companies. As mentioned, companies are structures employing a wide spectrum of workers with respect to demographic characteristics, professional experience, family and social relations. Thus targeting company workers may be seen as an opportunity to reach a much wider population of subjects. “Care International” in Ecuador has a program in occupational settings implementing policies to avoid discrimination against HIV positive workers but no studies have been conducted to assess knowledge about HIV/AIDS transmission and prevention measures. It has been working with 470 companies in the 17 most important cities of the country; as a result, in 2009, 54% of these companies had implemented nondiscrimination policies [12]. In 2007, the International Labor Organization implemented a program in Peruvian companies to enhance knowledge regarding HIV transmission but the results of the intervention are not available yet [13]. In Chile, the study “HIV/AIDS knowledge and occupational risk in primary healthcare workers from Chile” evaluated the level of knowledge about HIV transmission and reported that 63.8% of healthcare workers had an appropriate level of knowledge, which is 10% more than the subjects in the present study [14]. Most studies implemented in other Latin American countries with similar socioeconomic profiles as Ecuador were conducted in healthcare institutions among healthcare workers. A study among truckers (2009) in Morobo, Sudan (n = 300 aged 15–50 years old) reported that HIV prevention measures knowledge was very high (93% of condom use and 89% faithful with uninfected sexual partner). By contrast, their prevalence of HIV transmission knowledge was lower: 99.3% had misconceptions about HIV transmission by mosquito bites, 87.3% held incorrect beliefs about transmission by sharing a meal, however only 2.9% of respondents had the misconception that a healthy looking person cannot transmit HIV. The differences between HIV transmission and prevention measures knowledge is striking [15]. Another report in Sudan in the Lakes States (n = 3,326) registered that 36% of female subjects (15–49 years old) had accurate knowledge of HIV transmission and prevention measures, which is similar to the present study. However, more than 2% of Sudan’s population is HIV infected and the intervention programs are well developed [16]. A study among health care providers reported that 72% had HIV/AIDS general knowledge (HIV virology, transmission, symptoms, prevention strategies and risk assessment) in Tanzania [17]. More than 5% of adults in Tanzania were HIV infected and the disease seems to have stabilized over time. Another study in health care workers in Tamatave (Madagascar) reported a low HIV general transmission knowledge (18%) in 2002 [18]. In contrast to Tanzania, in Madagascar HIV cases increased every year but other factors such as low education, poverty, limited access to health and social services, high rates of partner change, and an increasingly transient population haven’t helped efforts to stabilize the epidemic.

The present study suffers from a number of limitations. The database of the “Superintendencia de Companías de Ecuador” does not contain all existing companies in the country, so that a substantial portion of the more informal companies has not been considered. In addition, despite a careful selection procedure, participation in the study was always on a voluntary basis. Therefore, the impact of factors such as education level, questionnaire fear or religious convictions is difficult to assess but should not excluded. Major efforts have been made to recruit the initial number of companies and hence to reach a large representative sample of workers, in particular by respecting the proportions of workers in the various job categories. It is clear that the material and financial resources of the companies and also their working sector (commerce, manufacturing or real estate) can influence the educational level of their workers. Therefore, the proportion of workers with a low education level in the study population may be notably underestimated. The study has been limited to three provinces; Pichincha, Guayas and Azuay. Guayas is one of the most populous provinces of Ecuador and its harbor, Guayaquil, is a main access point to outside world. Pichincha is the province of the capital, Quito, and a central commercial city in Latin America. Finally, the Azuay province with its capital, Cuenca, is more remote and close to the Amazonian region of the country, less exposed to HIV/AIDS progression. Thus, the three selected provinces should give a fairly good, although not perfect, picture of the country. Finally as already mentioned the working sectors considered in this study assemble the largest number of companies in Ecuador and also employ the greatest number of workers.

## Conclusions

The present cross-sectional study based on a stratified cluster sample of company workers demonstrates for the first time a major lack of HIV/AIDS knowledge in occupational settings in Ecuador. This is influenced negatively by factors such as older age, male gender, low education level, manual labor, and positively by married status and previous exposure to intervention programs. Based on these findings, HIV/AIDS intervention programs should be urgently needed in companies where no such programs have ever been held. Special attention should be paid to single manual labor workers aged 40 years or more and with low educational level. The group of younger workers more aware of the HIV/AIDS epidemics should also benefit from more specific recommendations as they are the most sexually active group. Finally, the study has demonstrated its feasibility in providing prevalence estimates of HIV/AIDS transmission and prevention measures knowledge in a large part of the active Ecuadorian population through the national company network. This may foster similar initiatives in other countries.

## Abbreviations

AIDS: Acquired immunodeficiency disease syndrome;CDC: Center for Disease Control and Prevention;CI: Confidence interval;HIV: Human immunodeficiency virus;IQR: Interquartile range;MPHE: Ministry of Public Health of Ecuador;MSM: Men who have sex with men;OR: Odds ratio;SAS: Statistical analysis system;SD: Standard deviation;UNAIDS: Joint United Nations Programme on HIV/AIDS

## Competing interest

The authors declare to have no competing interests.

## Authors’ contributions

MC carried out the design, implementation, and acquisition of data in the study and drafted the manuscript. MF has been involved in revising it critically for important intellectual content. AA participated in the design of the study, has been involved in revising it critically for important intellectual content and supervised the statistical analysis. TB has been involved in the implementation of the study and revising it critically for important intellectual content. ND performed the statistical analysis and contributed to data interpretation. All authors read and approved the final manuscript.

## Authors’ information

Maria del Carmen Cabezas, MD, is a PhD student at the University of Liège (Liège, Belgium) and a researcher at the School of Medicine of Universidad San Francisco de Quito, Ecuador; her expertise is in HIV prevention programs.

Marco Fornasini, MD, PhD in epidemiology is a full time professor and researcher at Universidad de las Americas, Quito, Ecuador; his expertise is in chronic diseases and infectious diseases.

Nadia Dardenne, MSc in mathematics, is a biostatistician in the department of medical informatics and biostatistics, University of Liège, Liège, Belgium.

Teresa Borja, PhD in Psychology is a full time professor and researcher at the Universidad San Francisco de Quito, Ecuador; her expertise is in sexology and HIV.

Adelin Albert, PhD in Statistics, is professor and head of the department of medical informatics and biostatistics, University of Liège, Liège, Belgium.

## Pre-publication history

The pre-publication history for this paper can be accessed here:

http://www.biomedcentral.com/1471-2458/13/139/prepub
